# A multi-disciplinary education process related to the discharging of children from hospital when the child has been diagnosed with type 1 diabetes - a qualitative study

**DOI:** 10.1186/1471-2431-10-36

**Published:** 2010-05-27

**Authors:** Lisbeth Jönsson, Inger Hallström, Anita Lundqvist

**Affiliations:** 1 Division of Nursing, Department of Health Sciences, Lund University, Lund, Sweden; 2 Division of Nursing, Department of Health Sciences/Vårdalinstitutet, The Swedish Institute for Health Sciences, Lund University, Lund, Sweden

## Abstract

**Background:**

Worldwide, insulin-dependent type 1 diabetes is one of the most frequently diagnosed long-term endocrine disorders found in children and the incidences of this diseased is still increasing. In Sweden the routines are, according to national guidelines, when the child is diagnosed with type 1 diabetes, the child and its family remains at the hospital for about two weeks. There is limited knowledge about how a diabetes team handles a child and its family from admission to discharge, therefore the purpose of this study was to seek a deeper understanding of how the diabetes team's parent/child education process works, from admission to discharge, among families with a child newly diagnosed with type 1 diabetes.

**Methods:**

Qualitative data collection was used. Four focus-group interviews, with a sample of three diabetes teams from different paediatric hospitals in the south western part of Sweden, were conducted and the data recorded on tape and then analysed using qualitative content analysis.

**Results:**

The results indicate that achieving a status of self-care on the part of the patient is the goal of the diabetes education programme. Part of the programme is aimed at guiding the child and its parents towards self-help through the means of providing them with knowledge of the disease and its treatment to enable the whole family to understand the need for cooperation in the process. To do this requires an understanding, by the diabetes team, of the individualities of the family in order to gain an overall picture.

**Conclusion:**

The results of this study show that the diabetes education programme is specifically designed for each family using the internationally recommended clinical practice guidelines with its specific aims and objectives. Achieving the families' willingness to assist in the self-care of the child care is the goal of the parent education process. To achieve this, the paediatric diabetes specialist nurse and the diabetes specialist paediatrician immediately and deliberately start the process of educating the family using a programme designed to give them the necessary knowledge and skills they will need to manage their child's type 1 diabetes at home.

## Background

Type 1 diabetes is one of the most frequent long-term endocrine childhood disorders, with incidence rates increasing rapidly worldwide. The lack of metabolic control has significance for the risk of developing late diabetes complications [1]. Research has provided a substantial amount of evidence for the relationship between psychosocial factors and metabolic control [2-5]. The goal of the Saint Vincent declaration [6] and the International Society for Pediatric and Adolescents Diabetes (ISPAD) [7] is to work for optimal health, social well-being and a good quality of life for all children and adolescents with diabetes, emphasizing the importance of education which is appropriate to the age of the child and includes the family, school or college in the process.

Patients diagnosed with type 1 diabetes, not acutely ill, are treated either on an out-patient or in-patient basis. A questionnaire survey answered by PDSN's (paediatric diabetes specialist nurses) and DSP's (diabetes specialist paediatricians) made in the UK and involving 75 paediatric clinics, revealed that home care of children newly diagnosed with type 1 diabetes was practiced by 33% of the clinics [8]. Some eastern European countries mostly hospitalise children with diabetes irrespective of how serious their illness is [9] this also applies to Finland [10]. When a child is newly diagnosed with type 1 diabetes in Sweden, the normal routine is that hospital based care is prescribed and carried out according to the national guidelines [11] this involves about two weeks' stay in the hospital for the child and the parents. In Sweden, as around the world, the paediatric diabetes teams are multidisciplinary including PDSN, DSP, dieticians and counsellors and/or a psychologist. The PDSN is a registered nurse with training and expertise in diabetes and paediatrics and works as an educator, counsellor, manager, communicator and innovator, under their own responsibility [12,13]. Children, and their parents, are encouraged to be active members of the care team [14]. The goal for the children and their families is to manage the diabetes and the parents are given the main responsibility for the care of their diabetes sick child, depending on the child's age [11,7]. The definition of diabetes education, is according to Clement, [15] "The process of providing the person with the knowledge and skills needed to perform diabetes self-care, manage crises and to make lifestyle changes to successfully manage the disease" (p.1204). This definition was also adopted by the ISPAD [7]. At the initial phase it is important for the parents to be given information about the disease as well as continuously updated using written guidelines and information adjusted for the child's age and maturity [7,14]. However, it is not recommended to give detailed information about the disease to parents who are in shock [14]. Mol's [16] philosophy of the logic of care relating to a person, who has been diagnosed with type 1 diabetes, reveals that the parents of a child newly diagnosed with diabetes do not claim autonomy regarding the care of their child nor the implementation of the education programme about the disease and the management of the technical kit. This means that the individuation ("the family must learn to become someone different") called for by the logic of Mol's care philosophy is a material and technical detail of daily life. The parents do what they can to adapt themselves to a new lifestyle instead of continuing their earlier habits. They are unreservedly prepared to accept all the tools they are offered to make it possible for their child to live with diabetes (p 61).

Studies describing parents' experiences of having a child newly diagnosed with type 1 diabetes have shown that they found it devastating and difficult to handle [17-21]. The onset of the disease was usually undramatic. Often the parents visit the hospital thinking that their child had an infection, and became alarmed by the urgency shown by the health care staff and lost control since they were ill-prepared to deal with the sudden situation [22]. A Swedish study focusing on the experiences of each family member after their child was diagnosed with type 1 diabetes, found that the sick child and its siblings experienced sadness and anxiety after hearing the diagnosis and that the siblings were often kept out of the diabetes handling education process while the sick child remained in hospital [21]. Knowledge about how a diabetes team works with the families of a child newly diagnosed with type 1 diabetes from admission to hospital to discharge is generally limited [12]. Therefore the purpose of this study was to seek a deeper understanding of how the diabetes team's parent/child education process works, from admission to discharge, among families with a child newly diagnosed with type 1 diabetes.

## Methods

### Study design

As it is the diabetic team who cares for the family during the hospitalisation of a child newly diagnosed with type 1 diabetes; we used a focus-group design to obtain in-depth information directly from the diabetes team members. The focus-group was considered to be a natural environment for the team as they could share their experiences and be influenced by each other - just as in their daily work [23]. Three paediatric hospitals in the south western part of Sweden were contacted. One of the hospitals was a university hospital caring for 24 to 26 children, newly diagnosed for type 1 diabetes each year and the two other hospitals were county hospitals caring for 20 to 24 children annually. The hospital context is described in Table 1.

**Table 1:** Demographic characteristics and hospital context.

Characteristic	Hospital I Number	Hospital II Number	Hospital III Number	Age	Professional experience of diabetes care (years)	Total
Diabetes specialist paediatrician	2	1	1	45-49	16-20	4

Paediatric diabetes specialist nurse	2	2	2	45-49	16-20	6

Counsellor	1	1		50-54	6-10	2

Psychologist	1	1		45-49	6-10	2

Dietician	1	1		45-49	6-10	2

	7	6	3			N = 16

Number of children (0-18 years) diagnosed with type 1 diabetes	22-24 yearly	24-26 yearly	20-22 yearly			

Number of children (0-18 years) in the catchment area	Approx 54 000	Approx 70 000	Approx 64 000			

Policy for the average of the hospital stay	One week	Two weeks and one week day-care*	Two weeks			

* During the day care at the hospital the parents followed the child to day-care, school, or the after school centre. At lunch time they came to the day care ward for an appointment with the PDSN and if needed an appointment with one other diabetic team members to discuss any problems that arose.

### Participant recruitment

After having had contact with the PDSN's in the diabetes team at each hospital concerned, it emerged that each team consisted of a number of PDSN's, DSP's, a dietician, a counsellor and a psychologist. The recruitment of the diabetes team members, into the focus group interview, was conducted by the PDSN in each team. The PDSN was asked to convey verbal and written information about the study to the diabetes team and to give a form for informed consent to each of the participating team members. Informed consent was obtained from each participant. At one of the county hospitals the PDSN chose not to ask the dietician, the counsellor and the psychologist to participate in the study as these were not so much involved in care of the family during the initial hospital stay. The other PDSN's did not question the participation of any of the other team members in the interviews. The study was conducted according to the Helsinki declaration [24] and the purpose of the study, time commitment, confidentiality, and the participant's right not to participate and their right to discontinue participation at any time was explained verbally and confirmed in the written information. Permission to conduct the study was obtained from the chief physician at all three hospitals. Since the study involved professionals who were being asked to answer questions related to themselves and their own profession it does not fall under the Swedish Law regarding Ethical Testing in Research, referring to human beings [25] therefore approval from the research ethics committee was not applied for.

### Conducting the focus-group interviews

Four focus-group interviews with three to six participants were conducted during the autumn of 2008 and the spring of 2009. At one of the county hospitals there were two focus group interviews performed as all the diabetes team members could not be present due to an emergency occurring in connection with the planned interview. The first focus-group interview at that county hospital was attended by two PDSN's, one counselor, one psychologist and one dietician and the second interview included one PDSN and two DSP's. Although this was not ideal, important information was anyway shared in both interviews. From the second county hospital, two PDSN's and one DSP took part in the interview. At the university hospital two PDSN's, one DSP, one dietician, one counsellor and one psychologist were present at the interview. In total, 16 team members were interviewed at the three hospitals (Table 1). In order to allow the participants to share their views in an unaffected manner we choose a few areas of their work that we wanted illuminated. The areas were (i) cooperation among team members, (ii) communication within the team and with the family, (iii) the division of the work with the family among the team members and (iv) how families were involved in the education programme. These areas were described in the information letter given to the participants as preparation for the interview.

Each interview lasted between 60 and 80 minutes and started with an open question; "Please, describe how the diabetes team members at your hospital are working with the families of a child newly diagnosed with type 1 diabetes during the time they are in the hospital". The first focus-group interview was moderated by the third author (AL) and the first author acted as assistant moderator. The following three focus group interviews were moderated by the first author (LJ) assisted by the third author. The focus-groups discussions were taped. The participants were informed that the transcripts would not contain personal identifiers and that their participation was voluntary. During the focus-group discussion the moderator encouraged the participants to express their own perspectives and views and to respond to other team members' statements. The moderator asked supplementary questions in order to strengthen the content and to find if there were any additional issues that the participants wished to highlight. Finally, a check was made to see whether all the predetermined interview areas had been addressed. The assistant recorded the group dynamics and interactions, and added supplementary follow-up questions at the end of the interview [23].

### Analysis

All interviews were transcribed verbatim, three by the first author and one by a secretary. The method chosen for analysis was qualitative content analysis [26]. All data were analysed independently by the first and third author. First, the text of all the interviews was read repeatedly, in its entirety, to achieve an overall picture of the content and a naive understanding. After that, all the text was divided into meaning-units and later on condensed to catch the meaning in the units. The condensed meaning-units were coded and codes with similar content were amalgamated. After which, the codes were sorted into sub-categories and categories based on differences and similarities.

The interview text was read again to confirm that all text relevant for the purpose was included in the categories and subcategories which constituted the manifest content. In the next step all the authors discussed and reflected upon the tentative categories to find the latent content. The focus moved from what the family members need to know to how the diabetes team members try to inspire a sense of confidence and ability among the family members. The latent content of the categories was formulated into a main theme and sub-themes concerning the discharge process conducted by the diabetes team. Lastly, all three authors reread the interview texts and reached consensus regarding the theme and the sub-themes.

## Results

The participants included four DSP's, six PDSN's, two counsellors, two psychologists and two dieticians. Demographic characteristics and hospital context are described in Table 1.

The diabetes teams reported that the educational process, which aims to prepare the family for leaving the hospital, began as soon as the family was admitted. The analysis of the study created a theme "Achieving adherence to self-care" followed by five sub-themes; Creating knowledge through practice; Creating a desire among the parents and children to be cooperative; Capturing the diversity of the whole family; Achieving practical application by the medically unskilled family; Obtaining an overall picture of the family.

### Achieving adherence to self-care

During the initial hospitalisation of the child, the focus of the diabetic team was on teaching the family members to administer insulin, monitor blood glucose levels, regulate diet and be able to apply this knowledge in a relevant way. It was not expected that the family members would be totally competent in diabetes management by the end of hospitalisation. During the hospital stay, equal emphasis was put on encouraging parents and the sick child to actively participate in the care. Before discharge the family ought to show that they were willing to responsibly engage themselves in the care of their sick child and that they will work for the child's best interests and in close cooperation with the PDSN and the DSP.

#### Creating knowledge through practice

The PDSN and nurses on the ward tried, in a respectful way, to get the family members to focus on the diabetes management education programme and to try to replace their anxiety and distress by actively taking part in the child's care.

The education programme focused on differences in diet, how the blood glucose level varied and the administration of insulin. Parents were invited to ask questions. Frequently asked questions were, what the family's daily life would look like and how things would work for their sick child at school, etc. The PDSN was engaged in each family issue and revised and enhanced the family's understanding of the disease, on the basis of these issues.

*"When they [the parents] have the value of the blood glucose, I [the nurse] ask what they are thinking about (regarding) the amount of insulin dose. Even if you think, as staff, that it is the wrong amount of insulin, you let them "do" it and then you evaluate the situation together with the family. You try to give them [the tools for] reasoning." *(4)

A diabetes checklist in accordance with ISPAD 2009 (ISPAD clinical practice consensus guidelines) [27] was used for information, teaching and demonstration, as well as the practical skills the parents needed to learn. The parents did not have a copy of the checklist used by the professionals. They were expected be able to handle the insulin pen, give injections, note the effect of the insulin, monitor blood glucose levels and explain the insulin doses in relation to the level of blood glucose, activities, as well as diet. The checklist was used only by the ward nurses and the PDSN, but the PDSN had the main responsibility for implementing and evaluating it.

The experiences of the diabetes team members revealed that the hospital stay was used to cram all the necessary knowledge and skills into the parents with very little possibility of the family gaining a full understanding of how to care for their child's disease. Most team members felt sure that the knowledge given to the child and parents at the hospital would not be properly followed up once the child was back at home. The PDSN focused on the point that family members should return home with the confidence that they will be able to handle the situation and that they had the capacity to meet the demands that were to be put on them, without necessarily understanding the disease and the care of it as a whole.

*"The patient and their family should not become afraid in the beginning. It is very, very important to convey a feeling of hope and give them courage. The teaching and learning must be handled at their own pace and we [the staff] must recognise their pace of doing things"*. (1)

The diabetes team members all tried to inspire the newly diagnosed child with a feeling of pride and to try to help them to enjoy their time in hospital. One team member thought that the equipment used in the care could be a way of inspiring such a feeling. The team member's hope was that the child would find it "fun" to come home and to show the insulin pen and the blood glucose meter to friends and relatives.

It was important to plan for the diagnosed child to return home for a short leave early in their hospital stay. Before this leave the child either should have experienced a hypo-glycaemia incident while at the hospital, which mostly occurred. In rare cases a hypo-glycaemia was provoked after being prescribed by the DSP, it depended on the circumstances. The first home leave would be for some hours, then be extended to half a day, followed by spending the night and finally a weekend at home. Besides the necessary equipment for the diabetes treatment at home e.g. blood glucose meter and dextrose, during the first home leave and also subsequently insulin, the family was given the phone number to the PDSN. This was a way for the PDSN to give the parents some security and to test whether the parents and children (depending on age) were able to use the blood glucose meter and understand the value given for blood glucose when they were away from the hospital. If the PDSN felt that there was any doubt as to the parent's capacity to take the samples or that they were unable to cope, they would ask the parents to phone in the blood glucose value they had recorded and to ask any questions they might have.

*"It is important that the family convey to the nursing staff that they accept to gradually take on the responsibility [for the diabetes care]"*. (4)

After the period at home the parents and the child often had questions they wanted to discuss with the PDSN and ward nurses. These could be related to issues such as what food to buy, whether the child could sleep over with friends, how much the child must keep active and to any other common situations in everyday life. Advice and individual support was given which helped strengthen the capability of the family.

#### Creating a desire among the parents and children to be cooperative

The PDSN was the one who worked most with the family, followed closely by the DSP. These professionals were responsible for the continuity of the care. The PDSN is specifically responsible for the care of the family's needs, preferences and participation in the care of the child. The goal of the PDSN and the DSP was to establish a two-way relationship with parents and the child in the early stage of the disease. Therefore, PDSN's had planned and unplanned appointments daily, or four to five times during the family's hospital stay and the DSP's at least two appointments with the family during the same period. Both parents were encouraged, to stay at the hospital and were allowed to report sick in order to take care of their child. Siblings were encouraged to stay at the hospital so that family cohesion was maintained and the siblings could gain an insight into diabetes care. Sometimes even grandparents visited the family and were also offered information about the care of children with type 1 diabetes.

A trusting relationship between the family, the PDSN and the DSP was important due to the fact that the PDSN and the DSP were to be involved with the family for years to come. In order to achieve this relationship PDSN's and DSP's emphasised that while they were both experts in the care and treatment of diabetes they were also fellow human beings who could empathise with the emotional experiences of the whole family. This way they could form an opinion about whether the parents were really willing to take responsibility for their child's illness. Sometimes the PDSN and the DSP experienced that the family members had in fact a good knowledge and the necessary skills even though the parents had expressed the opposite. The support from the care staff was then about getting family members to trust in their own abilities.

*"We are not so authoritarian. The patient and the parents are, or must be, members of the team already in the initial phase of the disease. We let the patient know that he or she is the most important person in the team"*. (2)

A counsellor, psychologist and dietician were often not involved in the care of the family during the initial hospital stay. However, a family had often only had one short appointment with these team members where they were informed about what support they could expect to receive from them, if the need arose at any time. At that appointment the psychologist, and possibly the counsellor, discussed the ongoing situation within the family. The counsellor always gave information regarding the state financial benefits that were available to the family. The dietician took care of any special diet requirements the family had while staying at the hospital and always met the family shortly after discharge as was often it was only then that any dietary problems became apparent to them.

Close cooperation in the form of weekly meetings, between all the diabetes team members, ensured that the team members were always up to date regarding the status of each family with a child suffering from type 1 diabetes. During the weekly meetings the professionals would update themselves and offer each other necessary support. The diabetes team members worked in close proximity with each other and had daily consultations as required.

*"When its teamwork, it is important that everyone address and knows what the other team members say and do. It does not mean that you have meetings with the family along with other team members"*. (4)

#### Capturing the diversity of the whole family

Instructing the parents of children with type 1 diabetes hospitalisation was individualised and was based on the diabetes teams' own frame of reference. Each hospital's diabetes team was confident that their policies were the best and not negotiable, especially when it came to the length of hospitalisation. However, some teams expressed a positive view on outpatient care although they did not enforce it that much. The instructing was tailored to each family's individual needs and, to some extent, the child's age. There was no definite line of demarcation for when a child was able to participate in its own diabetes care, but experience had shown that most often children at around the age of ten years wish to take part in their own care. It was experienced that the child's self-esteem increased when they were given the responsibility for blood glucose monitoring and the administration of insulin. For children under the age of ten, the parents were the focus of the education programme although the children were involved as well. For teenagers the focus of training was on the young people themselves but still the parents had the primary responsibility. Teenagers often acquired knowledge rapidly but for them the implementation was more difficult. The diabetes team sometimes separated the teenager and parents in recognition of the desire by young people to be independent. The diabetes team emphasised the importance of teamwork within the family.

*"We [the staff] want them [the family] to be part of a team. Teenagers should not have to take all the responsibility themselves. They need support from their parents"*. (3)

In families where parents were divorced, both biological parents and step-parents were expected to take care of a child with type 1 diabetes, in order that they should fully understand the disease and the treatment. In these cases, both families were involved in training that took place during the hospitalisation of the child. The child's best interests were at the heart of the matter, the intention being that both the biological and step-parents would achieve the desired level of knowledge and skill in handling a diabetes sick child in the greater family.

*"Everyone is informed - both the old and the new family"*. (4)

The education programme was identical for both Swedish and non-Swedish speaking families. One problem noted was that it could be difficult to train immigrant families due to their sometimes specific desires and when an interpreter was required. In such situations the checklist had to be completed while the interpreter was present and the time available was sometimes too short for the family to learn at their own pace.

#### Achieving practical application by the medically unskilled family

The home leave schedule must be followed by all families and all areas of the checklist "ticked off" before the child is discharged. The checklist had two functions, namely the acquisition of practical and theoretical knowledge as well as material for evaluation. The experience of the results of parent training was that most families had, through instruction, gained an insight into the situations that could arise for a child suffering from type 1 diabetes and had learnt to apply their knowledge even when they do not fully understand the ramifications of what they are doing. The PDSN's experienced that this "rule based knowledge" was limited, and that knowledge and the ability to apply knowledge in a relevant way are two different things. Many tools available for diabetes care remained unknown to the parents. The evaluation function involved testing the parent's theoretical knowledge by asking them questions in order to verify that the parents and the child had acquired the knowledge the PDSN advocated. Another task was to verify that the parents had the ability to perform certain aspects of self-care. One example was to explain what ketones (ketoacidosis) are, where they come from and how to take care of a child in such a situation. Another example to verify the forward situation was to ask the family to talk about their plans for the coming days after their discharge from hospital, e.g. if the child was participating in any sport or if the family was planning a vacation. It was a way to find out how the family members were thinking and the reasoning behind their decisions. If the PDSN experienced that there was some uncertainty in their reasoning, she called the family in the evening or the day after discharge, to make an extra check, so that the child did not get caught up in an unsuitable situation. As a follow up method one of the PDSN's used a knowledge based test comprised of 30 questions in which all the questions should be answered by the parents and also the child if it was over ten years of age. Afterwards the PDSN discussed the answers with the family and filled in areas where knowledge was either incomplete or missing.

*"Just because you have the knowledge, it is not certain that you can put your knowledge into practice"*. (3)

#### Obtaining an overall picture of the family

The DSP's and PDSN's, implied that they had genuine concern for each family where diabetes was present and a desire to get to know the families. The overall picture of the family was achieved by observation of the family's non-verbal communication i.e. the parent's body language and how secure the parents and the child appeared to be in using the knowledge and skills they had been taught. Another way of achieving an overall picture of the family was to get to know them by discussing their daily life and interests before their child had become ill, with the purpose of being able to offer the best care for each (individual) family.

*"Children's well-being reflects how parents cope with the disease and care"*. (4)

Did the parents appear worried, how was their behavior towards each other when being discharged from the hospital, were all important points to observe. Such subtle signs guided the professionals about how comfortable the family really was with the burden of care they were about to bear.

*"It can be both words and when I [the DSP] see how the family is. Are they looking nervous and (do they) look at each other, and so on. It is like, a feeling"*. (2)

After a child's discharge, the PDSN was available on the phone during daytime for families to call and the diabetes nurses on the ward were available at nights and weekends.

## Discussion

This study has the intention of shedding light on the diabetes teams' education programme that takes place during the hospital stay for families with children newly diagnosed with type 1 diabetes. The study also fills the gap caused by the lack of empirical studies showing how the diabetes teams work with the families in practice [12]. An interesting result is, that regardless of whether the diabetes team's time aspect is one or three weeks, each family receives the same degree and content in their training related to handling the care of their type 1 diabetes diagnosed child. The length of a hospital stay for a type 1 diabetes diagnosed child varies between the hospitals, and each diabetes team was sure that their recommendation is the right one. International ISPAD's clinical practice guidelines [27] state; that it is recommended that diabetes survival skills are taught to parents and children as soon as the diagnosis is established [27,28]. The results of this study contains one main theme showing what the education programme is expected to lead to, and five sub themes showing of how the education programme is implemented.

Achieving the adherence to self-care by the parents, after their child's discharge, is what the hospital stay is expected to lead to. However we cannot say whether this is achieved but [21] findings demonstrate that it appears to be possible. Wennick and Hallström's [21] findings were that parents feel that they can rely on the staff and benefit from the PDSN's and DSP's encouragement that conveys to them a feeling that they will be able to manage the diabetes care at home. Achieving adherence to self-care is also a way of placing the child's best interests at the centre which is the goal in paediatrics [29]. It remains to be seen how the family members experience the education programme and its design.

The diabetes teams interviewed are all working in a similar way with the families, following the ISPAD clinical practice consensus guidelines i.e. the checklist [27,11,7]. The education at the hospital involves imparting knowledge through practice and bringing about a feeling of being able to manage the situation. Such motivation and enthusiasm brought about by the education programme encourages families to acquire further knowledge and skills, and is a method for creating better adherence to therapy [30] as well as improving the biomedical outcome [31]. The families are considered as being active members in the diabetic care team as is recommended in paediatrics [14,32,33,27] and are not given time to overcome the shock of the diagnosis before the items on the checklist are presented and the training begins. This is in line with Mol's [16] philosophy where she states that a family whose child has been diagnosed with type 1 diabetes do not feel good if they are overwhelmed with misery, instead it is better that the professionals put emphasize on that there are good treatments for diabetes these days (p 43), which is contrary to what is otherwise recommended when facing a crisis [32,33,14]. It is the PDSN who takes care of the families' uncomplicated crisis, which is also shown by others [34]. The findings reveal that there is a close cooperation between the psychologist, the counsellor and the PDSN, should the family need more extensive counselling.

DSP's and PDSN's discuss, together with each family, their newly acquired knowledge and skills with an open mind and in a receptive manner. Family centred learning is a way of capturing the diversity of family constellations by elucidating how the training is accepted and also the family's different needs, personal choices and learning styles [7]. The question that arises is; whether cramming knowledge into a family in a short space of time in the hope of ensuring correct application, at home, by persons who are not medically trained can be described as family centred learning. Mol's [16] stresses that this is the right thing to do. People with type I diabetes depend on modern technology for their survival. They die quickly without insulin; therefore professionals have to give (the parents) facts about the disease and the technologies available for care. First, after receiving the facts, can the patient assess his or her situation and come to a decision and act (p 12).

However, there seems to be a discrepancy in utilising the checklist with the families concerning the transfer of knowledge and the individualising of the education. The PDSN's are well aware that they are cramming a lot of knowledge into the family in a short time without the recipients necessarily being able to understanding the ramifications of it. However, they are also aware of the fact that it is first when returning home that the application of the knowledge needs to function properly. The cramming of knowledge may be interpreted as an urgent effort to transfer the diabetes survival skills recommended to be initiated as soon as the diagnosis is made [28]. Instead of attempting to cram the parents of the sick child with information and knowledge relating to how they are expected to handle their child's diabetes Mol [16] suggests that DSP's and PDSN's could offer the parents practical information and implement interventions at the request of the family. As the amount of information relating to the care of diabetes in a child is extensive it is the professional's responsibility to listen to the affected child's parents and make clear how much of the information related to the diabetes care they have absorbed and judge how they will manage and what additional help they may require. The role of the parents and any involved members of the family are to be fully aware of the situation as described by the professionals and to properly implement the knowledge and guidance they have received. Life patterns that the family had become accustomed to before the diabetes diagnosis of their child will require adjustment to the new situation and its effect on the family's life because caring is not simply implementing medical knowledge and technical help it also requires the commitment of all those involved to ensure that the implementation is effective and gives a satisfactory result [16].

As each PDSN and DSP has extensive experience of diabetes education and caring, they seem to be well aware of how to apply the education programme in a way that will attract the parents' attention and interest. One question arises regarding the fact that none of the families were given the checklist or any written plan specifically prepared for their child's care. The recommendation is that families with children with chronic diseases should have clear, written instructions and simple schedules. These care plans should be updated from day to day [35,36,7]. Llahan, Poulton and Coates [37] investigated the teaching methods, approaches and the tools used by the PDSN's in paediatric diabetes education and found that they all used verbal information usually backed up with leaflets or booklets. The fact that all of families in our study did not have access to the checklist could be a conscious strategic approach by the PDSN, since the relationship with the family is considered the main focus in developing interdependence. Further research on this issue is needed.

It seems that in our study, the care staff's experience was that all parents' wish to acquire the diabetes care knowledge offered so as to be able to take care of their sick child and participate in observing their child's condition and to be able to discuss their own observations proportionately to those of the staff. In paediatrics it is often said that parents' involvement varies from poor to active participation [38] but decision-making should involve the child the parents and one or more of the professional health care staff [32,33] and always with the child's best interest in focus [29]. It is suggested that further research be made to find out if it is common that parents of children newly diagnosed with type 1 diabetes are more motivated to care for their child, independent of inpatient or outpatient care.

The DSP's and PDSN's genuine concern for each diabetic family and the desire to get to know the families was manifested by their developing a close relationship with the parents in the hope that the parents would be encouraged to ask any questions they had related to the disease. DSP's and PDSN's are of the opinion that to establish such a relationship requires they use real empathy and sympathy in getting to know the family, not only concerning what they are saying and doing, but also in their approach as fellow human beings. This is in line with Carpentier, et al. [39] i.e. who note that directing the diabetes education and counselling efforts towards parents of newly diagnosed children might benefit from the care professionals paying attention to the parent's feelings of uncertainty so as to boost their self-efficacy for diabetes management.

There are limitations in our study. The key feature with focus groups is the active encouragement of group interaction among participants [40]. The recommendation is that participant group members do not know each other; this is in order to create dynamic interaction while being homogenous in the meaning that sharing will be influenced by differences in the characteristics of the participants [23] (p 72). However, focus-groups in existing groups raise challenges. One is to create an environment where the participants are willing to open share their concerns and suggestions [23] (p 172). Therefore all interviews took place in each teams working environment. In our study, the participant group members not only knew each other they had worked together for a considerable period of time. The recommendation is that five to ten people is the ideal number of persons to take part in focus-group interviews [23] (p 10). The number of members in our focus-groups varied from three to six. Smaller groups afford more opportunities to share ideas, but also result in a smaller pool of ideas [23] (p 10) and also can limit the group dynamics considerably [41] which was obvious in our study. The interaction during the interview(s) showed that each profession described their work with families while the other participants were more or less silent. Another contributory cause for the missed interaction can be that only DSP's and PDSN's are involved in the care of the family and the discharge process during the hospital stay. The DSP's and the PDSN's were in agreement with each other regarding the education programme so there were no confrontations between them. The non-confrontation also can be about that working in a diabetes team does not mean that the team members works together, but all team members is important for there to be a holistic approach to the families so that all family needs can be met in order that the family be able to adapt to the new diabetic lifestyle. The counsellor, dietician and the psychologist is consultants to the PDSN and the DSP. The purpose of this study was not to find out the relationship between the team members. We used a purposeful sampling, reflecting the procedures at both a university hospital and two county hospitals in order to enhance transferability and to minimise response bias. However, the transferability must be discussed in the light of different treatment regimes such as in- or outpatient care in different countries. In spite of the limitations mentioned the method clarifies the diabetes team members' roles, how each of them works or does not work with the family regarding the education programme and how the families are cared for. The trustworthiness was confirmed by the fact that two of the authors performed the interviews and all the authors were involved in the analysis, regarding categorization and identification of the themes. The research process is explained and quotations are presented [42].

## Conclusion

When a child is diagnosed with type 1 diabetes and the family is admitted to hospital for a one to three week stay, an education programme to prepare the family for discharge began immediately. The education programme entails the immediate and deliberate exposure of the family to knowledge and skills for handling the care at home of a type 1 diabetes diagnosed child by the DPSN's and the DSP's in a way quite different from the norm in other settings.

This approach requires further evidence to determine the outcomes for the family members i.e. what benefit and disadvantage has the family gained from of the education programme toward managing their child's diabetes after returning home. To achieve voluntary adherence to self-care by the parents the PDSN's and the DSP's involve themselves as experts on diabetes and as sympathetic fellow human beings. They are always available for the family and individualise the child's treatment based on their knowledge of the family's needs and emotional situation. There seems to be a paradox between the education programme and the care professional's relationship to the family. How these are joined for the benefit of the families is a matter to be explored further.

## Competing interests

The authors declare that they have no competing interests.

## Authors' contributions

LJ, IH and AL were responsible for the study conception and design and drafting of the manuscript. LJ and AL performed the data collection and the data analysis discussing the results with IH throughout the process. IH obtained funding and LJ and IH provided administration support. All the authors read and approved the final manuscript.

## Pre-publication history

The pre-publication history for this paper can be accessed here:

http://www.biomedcentral.com/1471-2431/10/36/prepub
